# Severe BCG immune reconstitution inflammatory syndrome lymphadenitis successfully managed with pre-antiretroviral counseling and a non-surgical approach: a case report

**DOI:** 10.1186/s12981-024-00614-7

**Published:** 2024-04-27

**Authors:** Percina Machava, Winete Joaquim, Joseph Borrell, Shannon Richardson, Uneisse Cassia, Muhammad Sidat, Alice Maieca, Cláudia Massitela, Yara Quelhas, Cafrina Mucuila, Beatriz Elias, Massada da Rocha, H. Simon Schaaf, W. Chris Buck

**Affiliations:** 1 Hospital Central de Maputo, Maputo, Mozambique; 2 Universidade Eduardo Mondlane Faculdade de Medicina, Maputo, Mozambique; 3 University of California Los Angeles David Geffen School of Medicine, Los Angeles, USA; 4 Desmond Tutu TB Centre, Department of Paediatrics and Child Health, Faculty of Medicine and Health Sciences, Stellenbosch University, Cape Town, South Africa

**Keywords:** IRIS, BCG, Lymphadenitis, HIV, ART

## Abstract

**Background:**

Bacillus Calmette-Guérin (BCG) reactions are the most common cause of immune reconstitution inflammatory syndrome (IRIS) in HIV-positive infants who initiate antiretroviral therapy (ART). There is limited evidence regarding the incidence of BCG-IRIS; however, reports from outpatient cohorts have estimated that 6–9% of infants who initiated ART developed some form of BCG-IRIS within the first 6 months. Various treatment approaches for infants with BCG-IRIS have been reported, but there is currently no widely accepted standard-of-care.

**Case Presentation:**

A 5-month-old male HIV-exposed infant BCG vaccinated at birth was admitted for refractory oral candidiasis, moderate anemia, and moderate acute malnutrition. He had a HIV DNA-PCR collected at one month of age, but the family never received the results. He was diagnosed with HIV during hospitalization with a point-of-care nucleic acid test and had severe immune suppression with a CD4 of 955 cells/µL (15%) with clinical stage III disease. During pre-ART counseling, the mother was educated on the signs and symptoms of BCG-IRIS and the importance of seeking follow-up care and remaining adherent to ART if symptoms arose. Three weeks after ART initiation, he was readmitted with intermittent subjective fevers, right axillary lymphadenopathy, and an ulcerated papule over the right deltoid region. He was subsequently discharged home with a diagnosis of local BCG-IRIS lymphadenitis. At six weeks post-ART initiation, he returned with suppurative lymphadenitis of the right axillary region that had completely eviscerated through the skin without signs of disseminated BCG disease. He was then started on an outpatient regimen of topical isoniazid, silver nitrate, and oral prednisolone. Throughout this time, the mother maintained good ART adherence despite this complication. After 2.5 months of ART and one month of specific treatment for the lymphadenitis, he had marked mass reduction, improved adenopathy, increased CD4 count, correction of anemia, and resolution of his acute malnutrition. He completely recovered and was symptom free two months after initial treatment without surgical intervention.

**Conclusions:**

This case details the successful management of severe suppurative BCG-IRIS with a non-surgical approach and underlines the importance of pre-ART counseling on BCG-IRIS for caregivers, particularly for infants who initiate ART with advanced HIV.

## Background

Mozambique ranked second in the world for new annual pediatric HIV infections in 2019 [1]. Despite the risks of Bacillus Calmette-Guérin (BCG) vaccination in immunocompromised patients, Mozambique still routinely vaccinates all infants, including those known to be HIV-exposed with BCG at birth due to high incidence of tuberculosis (TB) [2–4]. This public health approach to maintain good BCG vaccination coverage despite not having implemented virologic testing at birth for HIV-exposed infants is recommended by the World Health Organization (WHO) [3].

Infants living with HIV with previous BCG vaccination who initiate antiretroviral treatment (ART) are at risk for developing BCG-related immune reconstitution inflammatory syndrome (IRIS). BCG-IRIS presents after ART initiation and is a reactivation of *Mycobacterium bovis* BCG either locally or systematically, with higher risk of severe and disseminated disease in infants with greater baseline immunologic suppression [5]. While the exact incidence of BCG-IRIS remains unknown, studies of outpatient cohorts reported that 6–9% of infants developed BCG-IRIS, with 88% of cases occurring within the first two months of ART initiation [6, 7]. Of all IRIS cases in HIV-positive infants, approximately half are likely caused by BCG [8].

There is currently no widely accepted standard-of-care treatment for BCG-IRIS. While some studies have reported that spontaneous resolution without specific treatment is common in mild cases, other studies have reported on aspiration, local instillation of isoniazid (INH), silver nitrate, and surgical excision as possible treatment approaches [9, 10].

## Case presentation

On March 4th, 2022, a 5-month-old male infant was admitted to the infant ward at Hospital Central de Maputo (HCM) with diagnoses of oral candidiasis, moderate anemia, and moderate acute malnutrition. He had been previously treated with nystatin at a local healthcare center without improvement. His immunizations were up to date per the Mozambique national schedule, including the BCG live-attenuated vaccine given in the right arm deltoid area at birth in South Africa. He had age-appropriate psychomotor development and maintained a diet of exclusive formula feeding since birth. His weight was 6 kg, with weight-for-age Z-score of -2, and weight-for-length Z-score of -2.2, indicating moderate acute malnutrition. The patient had no known TB exposures. His mother was a 29-year-old woman, diagnosed with HIV at her five-month antenatal visit and started ART at that time. Per the mother’s report, the patient received a DNA-PCR HIV test at one month of age in South Africa during November 2021. However, she returned to Mozambique before receiving the results. His initial treatment upon admission included therapeutic milk formula, fluconazole, and ceftriaxone.

Due to high suspicion of HIV and no record of previous test results from South Africa, a point-of-care nucleic acid test (PoC-NAT) was performed on March 7, 2022, which yielded a positive result, and was subsequently confirmed. He was given a WHO clinical HIV staging of III due to oral candida, anemia, and moderate acute malnutrition. Laboratory results of significance at this time included an absolute CD4 count of 955 cells/µL (15%) and a negative Xpert MTB/Rif® test performed on a gastric aspirate specimen. Admission full blood count had a total white blood cell count of 8,700/µL (neutrophils 21.3%; lymphocytes 57.0%), a hemoglobin of 9.7 g/dL, and a platelet count of 412,000/µL, He was started on the Mozambique standard pediatric first-line ART regimen (abacavir/lamivudine + dolutegravir dispersible tabs) on March 15, 2022 after inpatient extensive pre-ART counseling which included education on the signs and symptoms of BCG-IRIS. After 11 days of clinical improvement, he was discharged on ART, prophylactic cotrimoxazole, a multivitamin-anemia supplement, and follow-up scheduled at HCM.

On April 4th, three weeks after ART initiation, he presented to the emergency department at HCM with a one-week history of subjective intermittent fevers and progressive right axillary lymphadenopathy. Physical examination revealed a small ulcer over the right deltoid region and an enlarged right axillary lymph node without fluctuance or inflammatory signs. The child was then admitted for an inpatient work up. He was feeding well, had no cough or fever, and had no other complaints. The following day, he was discharged with a diagnosis of localized BCG-IRIS lymphadenitis without signs of disseminated BCG disease. The mother was advised to follow up as an outpatient until the resolution of the lesion while continuing ART, prophylactic cotrimoxazole, and the anemia-vitamin supplement.

**Fig. 1:**
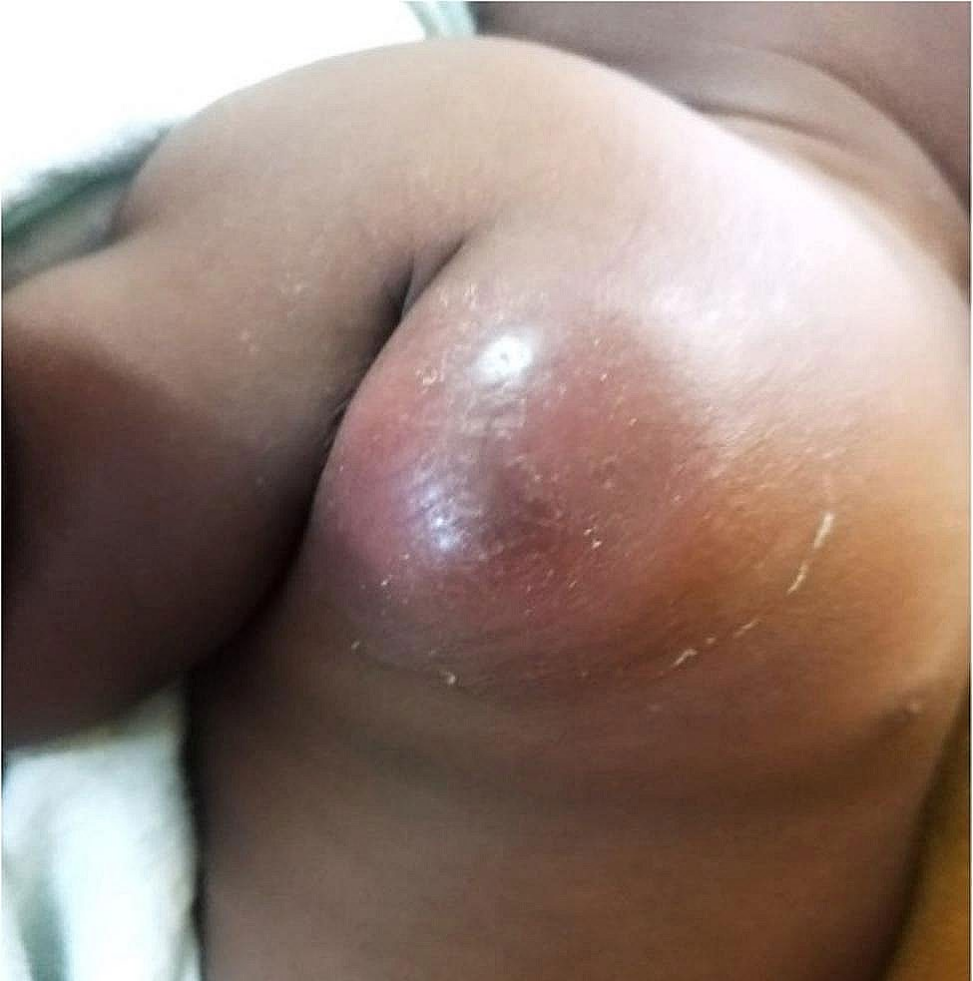
April 14th, 2022, ten days after initial presentation, and one month after ART initiation. Progression to localized inflammation and erythema with possible localized superinfection in the right axillary region.

On April 14th, the patient returned with an enlarged, inflamed, and tender right axillary adenitis without fluctuance, spontaneous drainage, or signs of systemic BCG disease (Fig. 1). He was treated as an outpatient with paracetamol, ten days of oral prednisolone (2 mg/kg/day), and seven days of amoxicillin/clavulanate for possible bacterial superinfection.

**Fig. 2:**
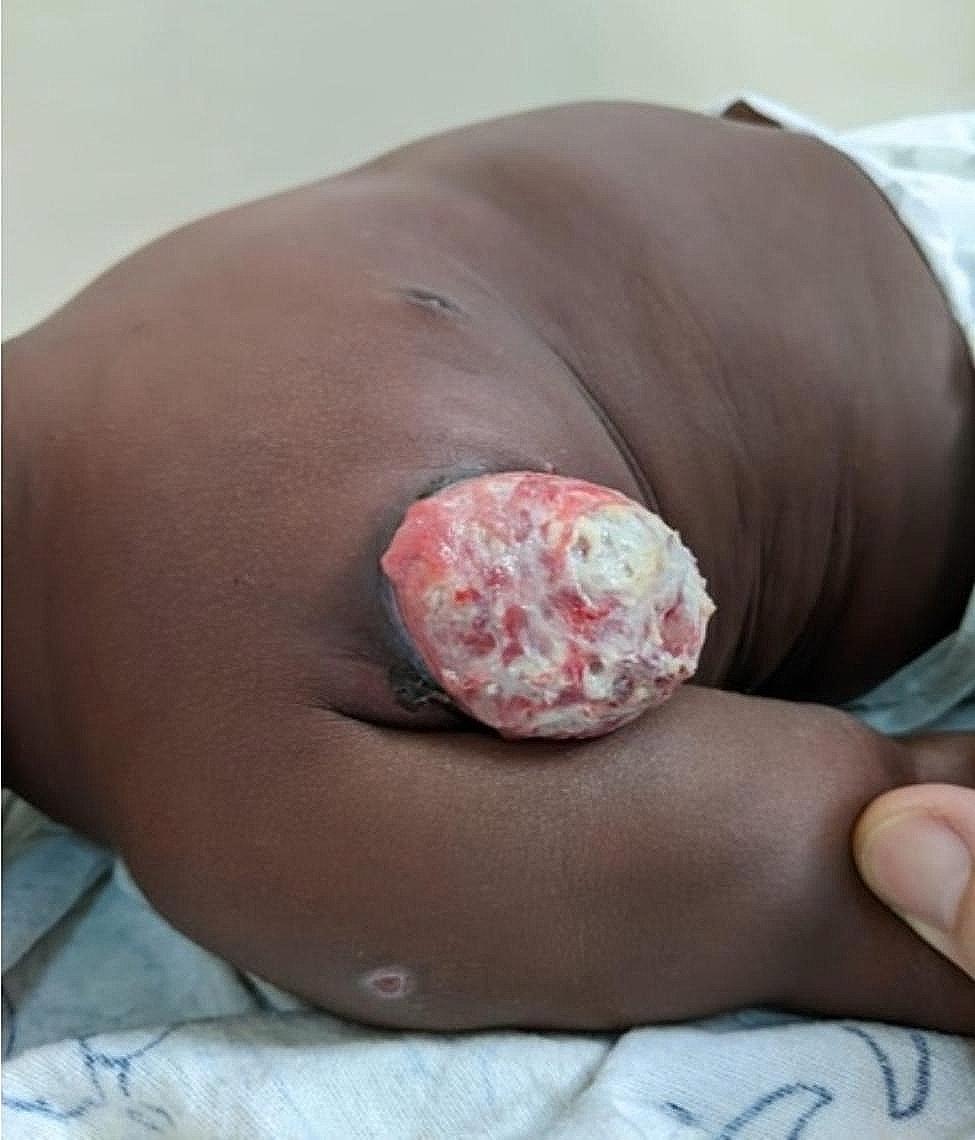
Right axillary nodal evisceration and small right deltoid ulcer, seven weeks after ART initiation.

On May 5th, at the following visit three weeks later, the child presented with five days of a completely eviscerated right axillary adenitis without fever or pain (Fig. 2). The mother confirmed good ART adherence since initiation. His laboratory results at this time were notable for an improved absolute CD4-count of 2,035 cells/µL (26%). The RNA-PCR viral load at this visit, which was less than two months since ART initiation, was 9,351 cp/mL with no baseline comparison since viral load testing is not routinely performed in Mozambique for new ART initiations. Other laboratory results were a white blood cell count of 18,100/µL (neutrophils 32.3%; lymphocytes 58.6%) and a hemoglobin of 9.4 g/dL. Pediatric surgery was consulted at this time and recommended against surgical intervention in favor of outpatient medical management due to concern about the increased risk of complications given the location and size of the mass. They advised treatment with topical silver nitrate with outpatient follow-up. After an additional consultation with a childhood TB expert in South Africa on May 17th, he was also treated topically with crushed INH tablets made into a paste while continuing silver nitrate. INH was initiated despite possible low-level INH resistance depending on the strain of *M. bovis* BCG he received with his birth BCG vaccine [11].

**Fig. 3:**
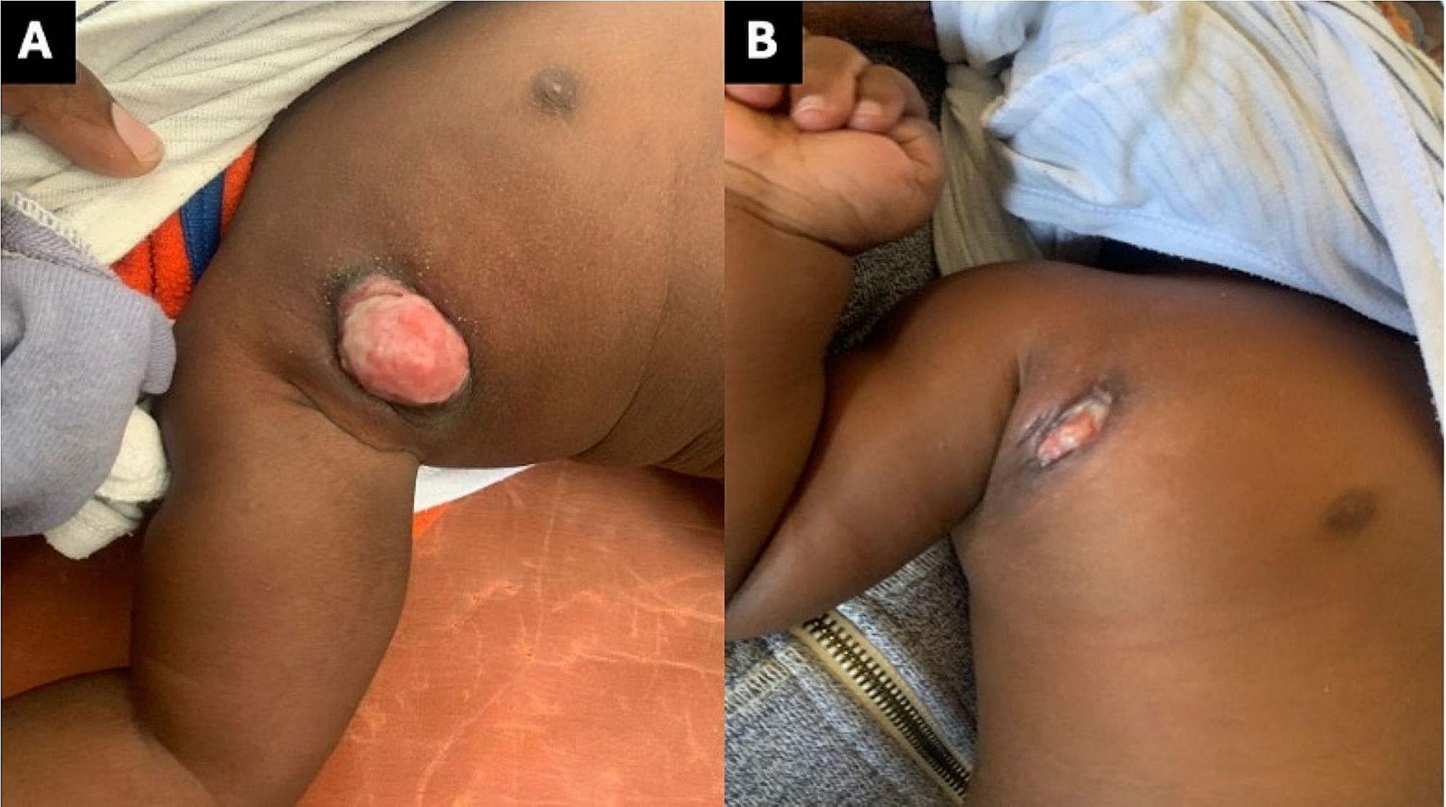
Improved right axillary region suppuration and inflammation one month after treatment with topical isoniazid and oral prednisolone (**A**). Substantially improved inflammation with healing wound (**B**).

**Fig. 4:**
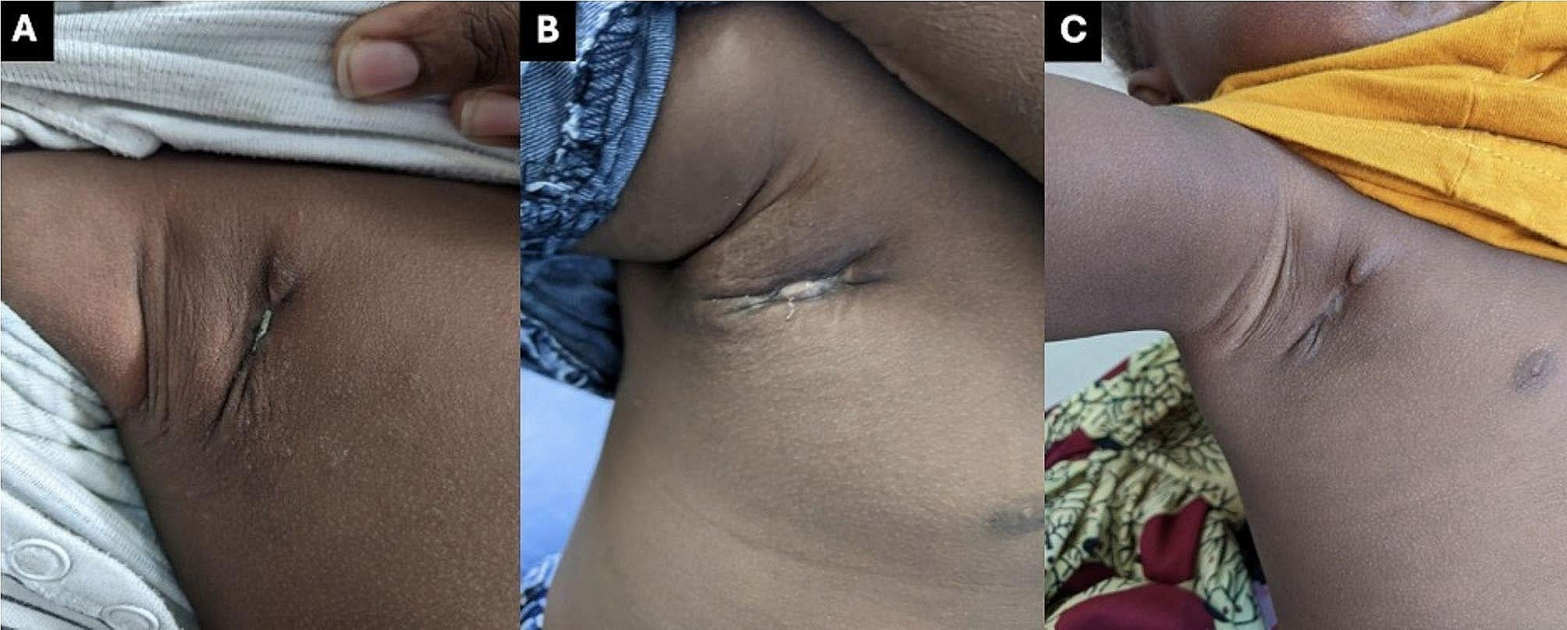
Progression of wound healing during follow-up from July 4th (**A**), July 15th (**B**), and August 15th (**C**).

At a follow-up visit on June 3rd, approximately 2.5 months since ART initiation and two months after initial presentation with BCG-adenitis, there was a good clinical response to treatment with marked mass reduction, improvement of adenopathy and erythema, and correction of anemia (Fig. 3A). Despite improvement in the adenopathy, prednisolone 2 mg/kg/day was prescribed to further reduce the inflammation. On June 16th, he had further clinical improvement (Fig. 3B). At this visit, prednisolone treatment was continued. On July 4th, he weighed 8.2 kg (weight-for-length Z-score of 1.8), was asymptomatic, and had a largely healed lesion (Fig. 4A). A two-week taper of prednisolone was initiated, and the mother was advised to continue treatment with topical INH and silver nitrate. The patient’s wound was entirely closed on follow-up on July 18th at which time the topical treatments were discontinued, with complete recovery noted at a follow-up consultation on August 15th (Fig. 4B and C).

## Discussion

IRIS has been described as a manifestation of immune recovery against latent pathogens upon restoration of cellular immunity through ART. Previous studies have demonstrated that possible triggers for IRIS may include infectious agents, host antigens, or tumor antigens, which may lead to atypical presentations making it difficult to recognize [4]. Children who are ART-naïve and received the BCG vaccine are at risk of BCG-IRIS upon initiation of ART, which may present at the primary injection site with or without ipsilateral axillary lymphadenitis [8, 12, 13]. The diagnosis is typically clinical without the need for confirmation of mycobacterial/BCG infection from aspirated pus or tissue biopsies [14]. Studies have reported that 6–9% of HIV-positive infants developed BCG-IRIS within the first six months of starting ART, with 88% of cases occurring within the first two months of ART initiation [6, 7]. The risk of BCG dissemination in immunocompromised infants not receiving ART may be high and particularly lethal, with a mortality rate of up to 75% in those with distant and disseminated BCG disease [5].

This patient’s presentation of lymphadenitis progressing to suppuration is similar to previous reports of BCG-IRIS in HIV-positive infants [6]. However we did not find reports of complete nodal evisceration documented in the literature. It has been reported that nearly half of all lymphadenopathies in BCG-IRIS and TB-IRIS will progress to suppuration [6]. While many mild cases of BCG lymphadenitis will resolve spontaneously in 4–6 months without any treatment, there are no widely accepted treatment algorithms for patients with more severe disease [8–10, 15].

The patient in this case was treated with sustained ART in addition to oral prednisolone, topical silver nitrate, and crushed INH tablets. Conclusions cannot be drawn about the efficacy of these various treatments from a single patient, but a discussion of the possible mechanism of action and therapeutic benefits of each is important. While no trials have determined the exact dose recommended for prednisolone in treatment of IRIS, common practice is to use prednisolone 1-2 mg/kg or equivalent corticosteroid for 1–2 weeks, followed by an individualized taper for the treatment of severe forms of IRIS [16]. Silver nanoparticles have been shown to have antimycobacterial effects, and when administered alongside anti-TB drugs, including INH, they may have improved extracellular and intracellular antimycobacterial activity [9, 10, 15, 17, 18]. We found no literature on the efficacy of crushed INH tablets as a topical treatment, but a review did report on the rapid resolution of symptoms when para-aminosalicylic acid (PAS) is applied externally on draining TB sinuses [19]. The pediatric TB expert in South Africa who was consulted for this case recommended using crushed INH based on similar positive results for patients with draining TB abscesses. In summary, we believe the treatment factors contributing to the rapid resolution of severe symptoms within three months were likely multifactorial.

Needle aspiration has been shown to be a possible treatment approach to BCG lymphadenitis when abscesses are present, shortening the duration of healing, decreasing the severity of complications, and reducing the risk of keloid scar formation [14]. However, the patient in this case did not have a fluctuant abscess amenable to drainage. Additionally, research has shown that surgical excision is a possible treatment approach in immunocompetent infants with BCG reactions but often requires general anesthesia in young infants [9, 14]. Access to surgical care is often limited in the sub-Saharan African countries where most at risk for BCG-IRIS live, and it is noteworthy that the severe localized disease in this case was successfully treated with a non-operative approach.

In this case, the patient presented with severe immune suppression at the time of ART initiation, suggesting he had a delayed diagnosis. It is likely that his DNA-PCR test performed at one month of age in from South Africa was positive, as he was never breastfed with low risk of post-natal HIV transmission. Immune suppression and high levels of HIV viremia at the time of ART initiation are known risk factors for the development of IRIS [6, 20–23], and studies have reported a reduction in risk of BCG-IRIS lymphadenitis when ART is initiated prior to immunologic or clinical progression [6]. The Mozambique national HIV early infant diagnosis algorithm calls for the first virologic test to be performed for exposed infants at one month of age, and the national vaccination schedule calls for BCG at birth regardless of the maternal HIV status. With these current guidelines, neonates born with HIV are receiving BCG vaccine and are at increased risk for developing disseminated BCG or BCG-IRIS after later ART initiation. Birth PoC-NAT for HIV has not yet been introduced in the country but represents an opportunity to identify newborns who were infected with HIV in-utero who would benefit from immediate ART initiation [24, 25]. However, the country would need to reconsider its policy of routine birth BCG vaccination, as the WHO also recommends that neonates and infants with confirmed HIV infection have BCG vaccination deferred until ART has been initiated and there is no severe immune suppression.

In this case, we believe the pre-ART counseling that included the possibility of BCG-IRIS lymphadenitis contributed to a timely diagnosis and successful treatment, as the mother returned for follow-up care when symptoms began and maintained good ART adherence throughout. On the breastfeeding ward at HCM, we see hospitalized infants who are more likely to have advanced HIV disease at the time of diagnosis and ART initiation, and anecdotally, the incidence of BCG-IRIS lymphadenitis that we see is much higher than the 6–9% reported from outpatient cohorts of infants [6, 7]. As a result, we routinely include an in-depth discussion of the signs and symptoms of BCG-IRIS adenitis with caregivers as part of our pre-ART counseling and emphasize that this complication is actually a good sign that the infant’s immune system is recovering. We believe this counseling has value in preventing families from discontinuing ART if BCG-IRIS occurs and recommend a similar approach for others initiating infants on ART.

In conclusion, BCG-IRIS lymphadenitis is a common complication after infant ART initiation in HIV-positive infants receiving BCG at birth. The patient presented in this case report had a severe presentation of BCG-IRIS adenitis with complete axillary lymph node evisceration, likely related to delayed HIV diagnosis associated with advanced clinical disease and severe immune suppression. We demonstrated that even severe localized BCG-IRIS adenitis can be successfully managed with comprehensive pre-ART counseling for caregivers that includes specific discussion of this condition to ensure sustained ART and a non-surgical approach, which is more feasible in many of the settings where infants with HIV receive care.

## Data Availability

No datasets were generated or analysed during the current study.

## Abbreviations

AIDS: Acquired immunodeficiency syndrome
ART: Antiretroviral treatment
BCG: Bacillus Calmette-Guérin
DNA: Deoxyribonucleic acid
HCM: Hospital Central de Maputo
HIV: Human immunodeficiency virus
INH: Isoniazid
IRIS: Immune reconstitution inflammatory syndrome
PCR: Polymerase chain reaction
PoC-NAT: Point-of-care nucleic acid test
RNA: Ribonucleic acid
TB: Tuberculosis
WHO: World Health Organization
